# Evaluation of patella height in native knees and arthroplasty: an instructional review

**DOI:** 10.1051/sicotj/2022037

**Published:** 2022-08-23

**Authors:** Christian Konrads, Anna J. Schreiner, Simone Cober, Daniel Schüll, Sufian S. Ahmad, Mohammad A. Alshrouf

**Affiliations:** 1 Department of Orthopaedic Surgery, University of Tübingen 72076 Tübingen Germany; 2 Orthopaedic Clinic Markgröningen 71706 Markgröningen Germany; 3 Department of Orthopaedic Surgery, Hannover Medical School 30625 Hannover Germany; 4 The School of Medicine, The University of Jordan 11942 Amman Jordan

**Keywords:** Patella height, TKA, Patella infera, Insall-Salvati, Caton-Deschamps, Reliability

## Abstract

Total knee arthroplasty (TKA) is the gold standard for treating advanced knee osteoarthritis. Among the postoperative complications of TKA are true patella infera (TPI) and pseudo patella infera (PPI), which should be differentiated since TPI exhibits significantly worse clinical outcomes. Multiple radiological patella height indices (PHI) exist; some were modified or originally designed for knees with implanted endoprostheses. However, there is no consensus on measuring and comparing patella height. Due to the lack of established, simple, reliable, and reproducible concepts for assessing patella height for arthroplasty, measuring patella height and the change of patella height by or after TKA have been challenging tasks for clinicians and researchers. This is a review of the current literature on methods for measuring patella height, with special attention to the ability to differentiate between the TPI and PPI after TKA. All literature on the topic was retrieved, and references from relevant articles were investigated until the end of April 2022.

## Introduction

The position of the patella is one of the most significant components for optimal knee joint function, and anomalies in patella height can impact knee function [1]. Two distinct problems have been described as a result of a misaligned patella in reference to the patellofemoral joint line: patella alta or high-riding patella in relation to the femur is associated with patellofemoral pain, recurrent patella dislocations, and Osgood-Schlatter’s disease [2]. Patella infera or an abnormally low patella is linked to a limited range of motion, joint pain, and crepitations after total knee arthroplasty (TKA) or trauma [3].

In recent years, patella height has become a significant concern in assessing knee problems and planning therapy, particularly following knee arthroplasty, since the number of TKAs performed every day has increased dramatically [4, 5]. Patella infera is one of the complications that might occur during or after TKA and is divided into two types. True patella infera (TPI) is characterized by a direct shortening of the patella tendon, most likely caused by scarring. Pseudo patella infera (PPI) is described as a change in patella height that is indirectly caused by the proximalisation of the femorotibial joint line (joint line elevation during TKA). This relative reduction in patella height in reference to the femoro-tibial joint line (knee in 0° of flexion) is common in TKA, and if it is severe, it can lead to poor functional results [6–8].

Several approaches have been reported for estimating patella height in normal knees and after the implantation of a knee endoprosthesis [9–15]. These approaches have advantages and disadvantages, as well as varying levels of reliability, reproducibility, and intra- and interobserver variability [16–18]. These approaches relate the patella position to the femur (direct evaluation) or the tibia (indirect assessment). Direct evaluation approaches are not generally used since they have been described as too difficult to implement [6]. The most extensively used radiographic methods are the Insall-Salvati (IS) [9], the modified Insall-Salvati (MIS) [10], the Blackburne-Peel (BP) [11], and the Caton-Deschamps (CD) [12] indices. The derived Caton-Deschamps index (dCDI) has recently been characterized by Konrads et al. as a simple and reliable method to evaluate patella height in knee arthroplasty postoperatively, and it can directly be compared to the original CD index used in the native knee before arthroplasty [13].

Patella height measurement is crucial in assessing knee conditions and can help with treatment planning [19]. Both TPI and PPI should be included in a comprehensive patella height evaluation. A reliable technique for assessing patella height and the change in patella height caused by TKA must be established for effective treatment planning. Developing concepts for assessing patella height is important to allow clinicians to distinguish between TPI and PPI postoperatively. The main objective of this review was to discuss radiological patella height indices for identifying the amount of TPI and PPI after TKA.

### The clinical relevance of patella height

Patella height is an important factor for the biomechanics of the whole knee, which is true for native knees and knee arthroplasty.

Patella height plays a role in patellofemoral instability. Patella alta contributes to instability because the patella will engage with the femoral trochlea only at higher degrees of knee flexion.

Patella infera can lead to limited flexion of the knee, to higher retropatellar pressures, and it can also affect active knee extension. It often leads to anterior knee pain.

True patella infera can develop by scarring after surgery even in the second postoperative year, but not later than 24 months postoperatively [16, 20, 21]. Pseudo patella infera due to proximalisation of the joint line during primary total knee arthroplasty and patella infera, in general, does not deteriorate the clinical outcome of primary total knee arthroplasty if the reduction of patella height does not exceed 10% [16].

With the osteotomy of the tibial tuberosity, a standard surgical procedure has been developed to directly influence patella height by distalisation or proximalisation of the tibial tubercle (bony insertion of the patella tendon).

### The history of patella height measuring

In the year 1938, Blumensaat firstly introduced a method for measuring the height of the patella on radiographs [22]. Since then, multiple different methods have been described; some of them were specially invented for utilization in arthroplasty.

Strict lateral radiographs should be used for measuring the patella height. Further, many indices are dependent on the knee flexion angle.

In Lyon, several works have been performed leading to the CD index, which can be used on lateral radiography or MRI. The CD index is rather independent of the knee flexion angle and can be used throughout a range of 10° to 80° of knee flexion [9, 12].

From MRI studies, it is known that the distance between the tibial plateau and the insertion of the patella tendon at the tibial tuberosity is rather constant, about 29 mm. A patella tendon length of more than 52 mm was considered patella alta.

Until 2022, no broadly accepted method or concept that has the common consensus of orthopedic surgeons and radiologists for measuring and assessing patella height in native knees and in arthroplasty.

## Materials and methods

**Figure 1:**
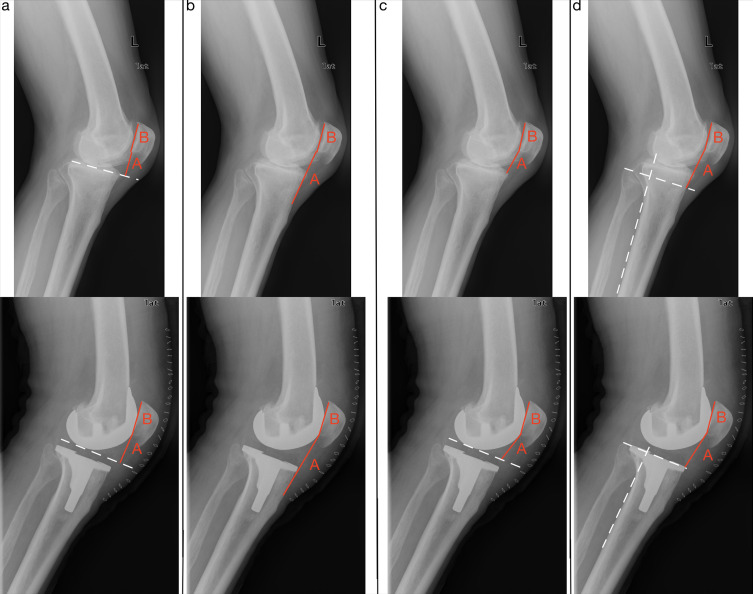
Indices for patella height measuring. (a) Blackburne-Peel index for native knee and modified Blackburne-Peel index for arthroplasty. (b) Modified Insall-Salvati index. (c) Caton-Deschamps index for native knee and derived Caton-Deschamps index for arthroplasty. (d) Modified Caton Deschamps index. Index value = *A*:*B*.

Figure 1 shows four different basic indices (BP and mBP, mIS, CD, and dCD, mCD) used for patella height measuring in knees without and with knee arthroplasty.

Some indices are femoral referenced, meaning they refer to the femoro-tibial joint line in the extended knee (direct evaluation), e.g., dCD index, mBP index. Some indices are tibial referenced (indirect assessment), e.g., IS index, mIS index, mCD index. The original CD and BP indices directly reference the joint line.

## Results

**Figure 2:**
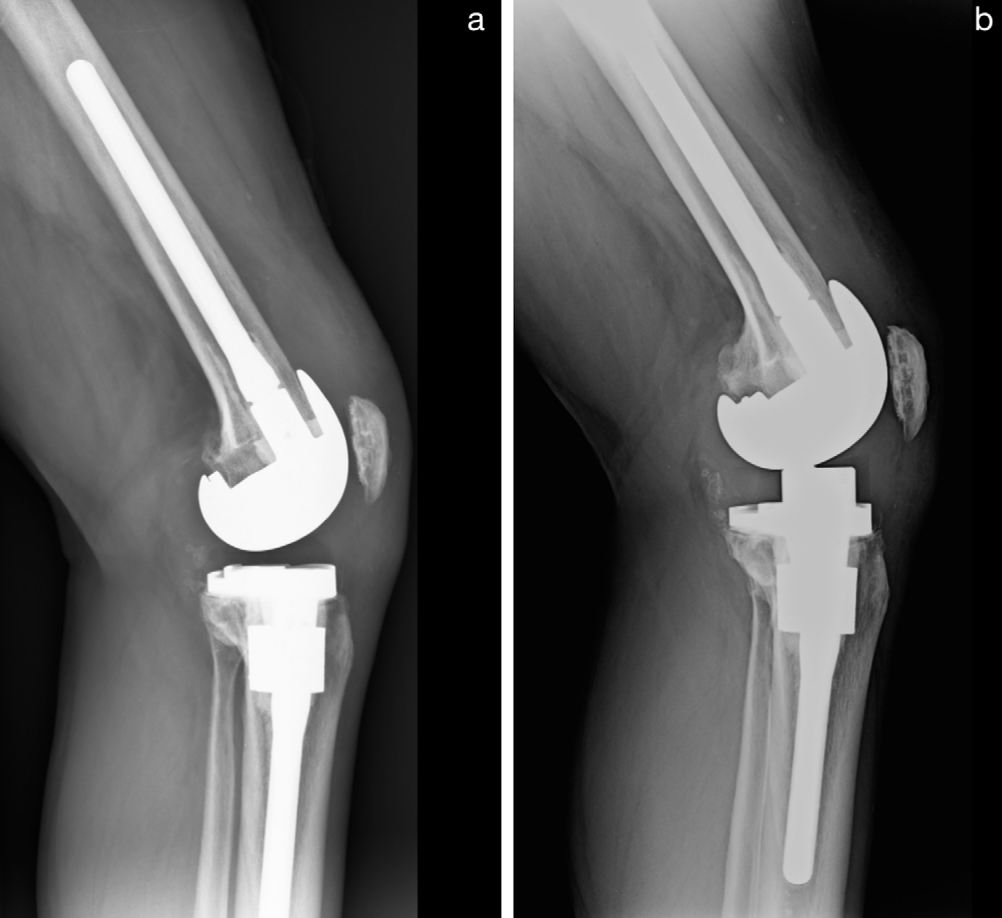
Knee arthroplasty before and after isolated tibial insert exchange to a thicker one due to preoperative instability. (a) Before insert exchange. (b) After insert exchange. Pseudo patella infera is created.

When the point of reference is tibial and not on the joint line but further distal, the value of the index is not altered, although a PPI might have been created by joint line elevation via insert exchange to a thicker one. Figure 2 shows such a case. The index values measured for this case are listed in Table 1.

**Table 1:** Patella height measurements before and after exchange of the tibial insert to a thicker one creating a pseudo patella infera.

Index	Before revision	After revision	Classification preop	Classification postop
Insall-Salvati (ISI)	1.2	1.2	Patella norma	Patella norma
Modified Insall-Salvati (mISI)	1.6	1.6	Patella norma	Patella norma
Modified Caton-Deschamps (mCDI)	1.4	1.4	norma?	norma?
Derived Caton-Deschamps (dCDI)	0.6	0.4	Patella norma	Patella infera
Modified Blackburne-Peel (mBPI)	0.7	0.5	Patella norma	Patella infera

Indirect measures with tibial reference like ISI, mISI, and mCDI remained unchanged. They measured true patella height (length of patella tendon) but could not detect PPI. The dCDI and the mBPI were able to correctly demonstrate the creation of PPI (Figure 2, Table 1).

## Discussion

### The need for a standard method

With a continuous increase in the number of TKA, determining the optimal method for measuring patella height after TKA has become paramount to optimizing the management postoperatively. In current daily practice, different imaging techniques and methods for measuring patella height are used [9–14]. Although the optimal method for measuring patella height after TKA is yet to be determined, we propose a standardized measuring concept based on current evidence, which could assist in the harmonization and comparability of clinical practice and future studies.

### The optimal standard method

Ideally, a method or concept of patella height measuring allows us to identify the amount of PPI and TPI contributing to patella infera, especially in the postoperative setting. The IS and MIS indices that use the anterior tuberosity of the tibia as a landmark are only able to detect the TPI, while the PPI will not be detected. Therefore, this mandated the use of additional methods of measurement, including BP or CD indices, to detect the PPI. Thus, surgeons recommended that in order to be able to distinguish between TPI and PPI, a combination of methods should be used [16, 17, 23, 24]. In addition, it should be highlighted that because TKA is more strongly associated with joint line elevation than other surgical procedures, the CD and BP indices are essential in the diagnosis of patella infera following TKA [24].

In a study on 92 primary TKAs, Konrads et al. evaluated the patella height and its correlation with clinical outcome. They demonstrated that patella height could be accurately assessed using the ISI and the CDI preoperatively, with the utilization of the ISI and the dCDI postoperatively [16]. Alternatively, the BPI or mBPI can be used instead of the CDI or dCDI. Moreover, good intra- and interobserver reliability for each of these indices were found. However, the sample size was limited, which reduced the power of the study and increased the margin of error, which may produce inconclusive results.

In another study by Xu et al., which included 256 patients who received primary TKA, it was demonstrated that while both the IS and MIS indices are adequate for evaluating patella height, the use of the MIS index postoperatively may be a preferable choice [21]. In addition, they recommended the use of the CD index preoperatively and BP postoperatively to detect PPI. The total number of positive cases was just 26 in this study. This limits our ability to reach a definitive conclusion on the reliability of these methods.

In a recent study, a new approach for evaluating patella height and the position of the joint line before and after total knee arthroplasty was reported, which included the use of axis-patella, joint axis-patella indices, and the joint line height [4]. This method showed comparable intra- and interobserver differences with the commonly used methods, showing that the new method is reliable. However, since this is the first study to describe this novel approach, further research is required to possibly support the scientific rigor and measurement validity of the study.

There are several factors to consider when measuring the patella height, but the reproducibility and accuracy of the ratios used are one of the most crucial aspects. The IS index demonstrated excellent reliability and has the best reliability of all the ratios, whereas the MIS index demonstrated moderate to excellent reliability, still being among the most reliable patella height indices [14, 25–27]. Similarly, a recent study showed that the most reliable technique of measuring patella height on conventional radiographs is the IS ratio compared to BP, CD, and MIS [28]. The high reliability of the IS index can be explained by the fact that it is easy to identify the superior and inferior ends of the patella joint surface on plain radiographs used for this index. However, the ICC for the IS index was lower postoperatively, possibly due to difficulty identifying the tibial tuberosity [29]. In contrast, Seil et al. and Berg et al. reported that the BP ratio is the most reliable [18, 30]. In addition, a review by Philips et al. in 2010 stated that BP and CD indices were the most reliable ratios [31]. Finally, an important factor to consider is the level of experience of the observer, with orthopedists and radiologist specialists having the highest interobserver agreement compared to orthopedic residents [25].

Clinical factors such as the knee flexion angle and quadriceps action are the key factors to consider when evaluating patella height ratios since they might have an impact on the accuracy of the assessment [32]. Furthermore, it is important to keep in mind while interrupting the results of the patella height measurement that there could be a difference in the normal reference range between cultural groups, as shown in a study among southern Chinese, Indian, and Middle Eastern populations since the reference range are based primarily on studies on western populations [33–35].

When deciding on a standard concept to analyze patella height in daily practice routine and science, a point of major importance is that the concept should be easy to measure in a reasonable time, and the chosen indices can be used preoperatively and postoperatively without and with implanted implants like partial or total arthroplasty of the knee. The CD index is accepted to be independent of the amount of knee flexion or extension. It can be used with native lateral radiography or MRI of the knee.

**Figure 3:**
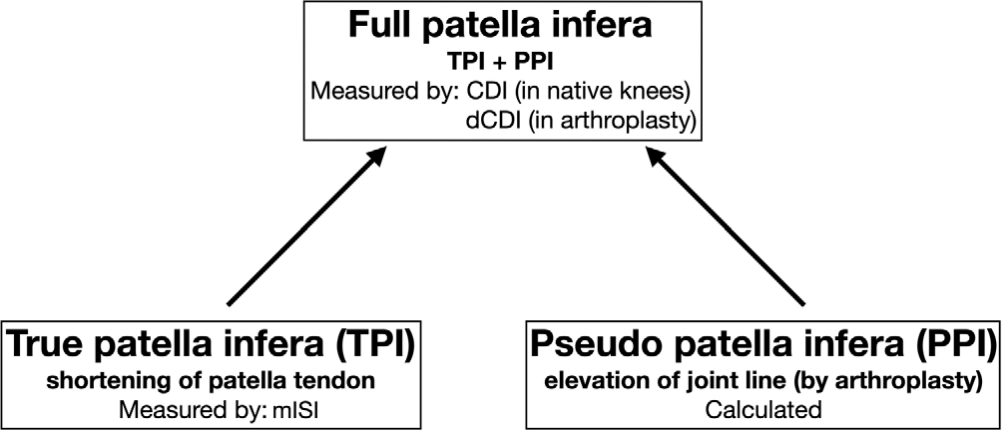
Definitions, reasons, and modalities of assessment of true and pseudo patella infera. CDI, Caton-Deschamps index; dCDI, derived Caton-Deschamps index; mISI, modified Insall-Salvati index.

**Figure 4:**
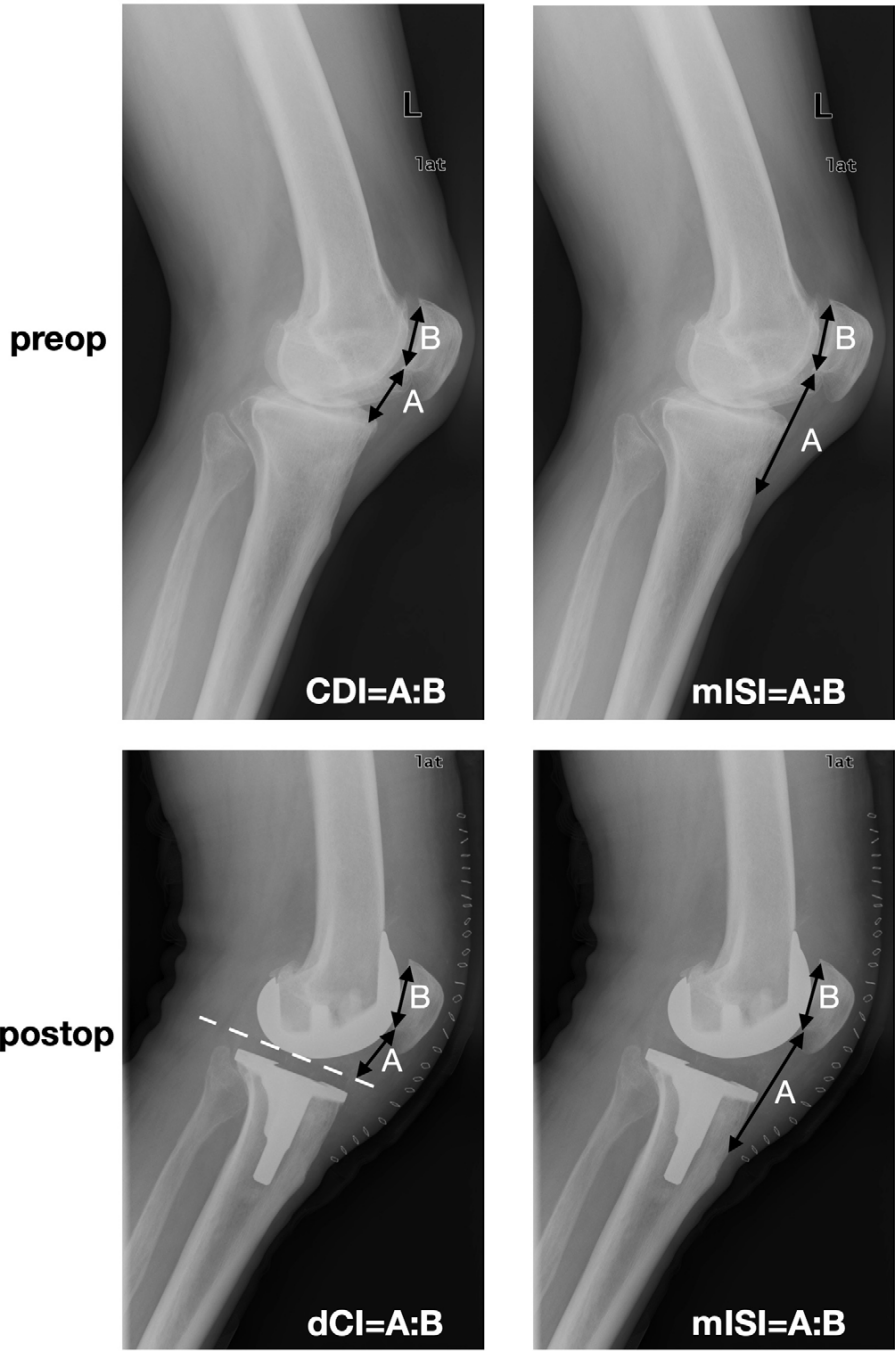
Concept of patella height assessment. Full patella height (CDI for knees without arthroplasty and dCDI for knees with arthroplasty implanted) and the amount of true patella height (mISI for all knees) are determined. CDI, Caton-Deschamps index; dCDI, derived Caton-Deschamps index; mISI, modified Insall-Salvati index.

Overall, the concept described and illustrated in Figures 3 and 4 seems to be the most practicable and comprehensive way to analyze patella height before and after knee arthroplasty or revision knee arthroplasty. The amounts of PPI and TPI contributing to the full amount of patella infera can be determined easily, knowing that CDI and dCDI identify full patella infera and the (m)ISI measures TPI only. Reference numbers for the classification of patella height in patella infera, norma, or alta are summarized in Table 2.

**Table 2:** Common radiographic indices used for classification of patella height.

Index	Patella infera	Patella norma	Patella alta
Insall-Salvati (ISI)	<0.8	0.8–1.2	>1.2
Modified Insall-Salvati (mISI)	<1.2	1.2–2.0	>2.0
Caton-Deschamps (CDI)	<0.6	0.6–1.2	>1.2
Derived Caton-Deschamps (dCDI)	<0.6	0.6–1.2	>1.2
Blackburne-Peel (BPI)	<0.6	0.6–1.0	>1.0
Modified Blackburne-Peel (mBPI)	<0.6	0.6–1.0	>1.0

### The drawback of current methods

The IS and MIS ratios do not change in cases of proximalization of the joint line following total knee replacement surgery; on the other hand, the BP and CD ratios show a change in the position of the patella articular surface in reference to the joint line, indicating the presence of a PPI [18]. The IS and MIS indices depend either on the length of the patella tendon or on the ability of the observer to identify the tibial insertion of the patella tendon at the tibial tuberosity. So, a standardized identification of the tibial attachment of the patella tendon is needed to avoid a significant variation in measurements [18]. Comparing preop and postop radiographs can help in this regard. In addition, the patella shape might result in a higher incidence of patella infera when utilizing the IS index, particularly in Cyrano patella (patella with a long, non-articulating inferior pole) [23, 36]. For the BP index, it is necessary to determine the tibial articular surface to draw a joint line. This can be difficult, especially if the radiograph is not perfectly lateral. Variations in the polyethylene inserts can cause additional difficulties.

### Limitations

Our present work has some limitations. Firstly, the narrative review design may be subjective and biased; however, to minimize publication bias, we conducted a literature search for current and historical publications, and every attempt was made to ensure that all relevant studies were included and discussed. Secondly, we did not consider non-English language studies. Thirdly, due to limited research, the optimal method to measure the patella height is not yet established. In the future, further investigations are needed to test the measurement method and validate it in greater depth.

## Conclusions

Finally, after reviewing the literature on this controversial subject and the currently available methods of measurements, the solution to detect the pseudo and true patella infera postoperatively with the highest accuracy is using the right combination of indices. In our opinion, the combination of Insall-Salvati and Caton-Deschamps or Blackburne-Peel preoperatively and the combination of Insall-Salvati and derived Caton-Deschamps or modified Blackburne-Peel postoperatively is the best concept.

It is critical to establish a gold-standard protocol that will enable surgeons to precisely identify surgical indications and will also allow us to compare clinical studies. In the absence of a comparison scale, the dilemma of choosing an ideal patella height ratio remains unanswered. However, the most important factor in measuring the patella height is using a combination of methods to avoid misdiagnosing the patient. Therefore, the concept proposed in the present instructional review seems sufficient and easy to use in daily practice and science.
